# UK CropNet

**DOI:** 10.1002/1097-0061(20000930)17:3<244::AID-YEA38>3.0.CO;2-P

**Published:** 2000

**Authors:** Jo Wixon

**Affiliations:** School of Biological Sciences University of Manchester Oxford Street Manchester M13 9PT UK

## Abstract

This review explores the UK CropNet site. The project is aimed at aiding the comparative mapping of cereal and other crop genomes. The site provides software tools for use by those working on genome mapping, and access to an array of databases that will be of interest to all members of the plant genomics research community, using several ACeDB interfaces. All screen views from the website are reproduced with the kind permission of Dr Sean May, Director, Nottingham *Arabidopsis* Stock Centre (NASC).


**Yeast**
*Yeast* 2000; **17**: 244–254.

**Website Review**

### UK CropNet
### http://ukcrop.net

Jo Wixon
*Managing Editor – School of Biological Sciences, University of Manchester, Oxford Street, Manchester M13 9PT, UK*

### Abstract

**This review explores the UK CropNet site. The project is aimed at aiding the comparative mapping of cereal and other crop genomes. The site provides software tools for use by those working on genome mapping, and access to an array of databases that will be of interest to all members of the plant genomics research community, using several ACeDB interfaces. All screen views from the website are reproduced with the kind permission of Dr Sean May, Director, Nottingham *Arabidopsis* Stock Centre (NASC). Copyright © 2000 John Wiley & Sons, Ltd.**

### Background

UK CropNet consists of project groups at the John Innes Centre (JIC), the Nottingham *Arabidopsis* Stock Centre (NASC), the Scottish Crop Research Institute (SCRI) and the Institute of Grassland and Environmental Research (IGER) (for details see **http://ukcrop.net/projects.html**). UK CropNet aims to produce databases and bioinformatic tools for the study of plant genome evolution. The site author is Keith Bradnam of the Nottingham *Arabidopsis* Stock Centre.

### Site structure

- About: Background on the *raison d'etre* of UK CropNet.
- Projects: A breakdown of the projects, with contact details for each group.
- Software: Tools available from the groups, mainly for comparative mapping.
- Databases: UK CropNet databases and mirrors of several US plant databases.
- What's New: News of improvements and update reports for the site.
- Help: E-mail address for queries and feedback.
- Links: Links to the home pages of the project teams and other related sites

### Site guide

### About

This section gives information on the scientific background of the project and explains the aims of UK CropNet with links to pages containing further information on each of the projects.

### Projects

This page has a section for each of the projects, with links to pages giving more information on the project and to the home pages of the host research centre. The names and contact numbers are given for the members of each group. A drop-down menu is provided at the top of the page for quick access to each project profile. Each project page gives background information on the plant genus or species under study and details the relevant resources that are made available in UK CropNet.

### Software

This page starts with links to information on new tools being developed by members of UK CropNet, such as CITA, a simple CORBA Interface to one or more ACEDB databases, and GFace, which is a general Java CITA client.

In keeping with the aims of UK CropNet, the other tools available on the site are mainly for comparative mapping.

A link to the source code for the Recombination Viewer tool is available, and a demonstration link takes you to a page explaining the concept behind the tool and the way the data is displayed. The tool is designed to help to assign ambiguous markers and to allow rapid detection of mainly heterozygous or mainly homozygous plants.

Grid Map is a Java version of the Oxford Grid

Copyright © 2000 John Wiley & Sons, Ltd.

traditionally used to plot orthologues between two species. Clicking on the icon link takes you to the relevant page of the John Innes Centre Bioinformatics website. Clicking the Grid Map link opens a Java applet window in which the program runs. To open a grid, use the 'file' menu to choose 'new grid'. A grid can be generated from the datasets available on the local server (following the instructions given on the webpage) or from a selection of databases (only zoodb is working at moment). Once the database is selected, a window appears to prompt the choice of two organisms and once this has been completed, the grid is drawn (Figure 1). There are several options for changing the appearance of the grid and it is possible to zoom in on a chosen cell. Clicking on a cell causes the 'cell properties' window to display the genes for which a point has been plotted in that cell. Clicking on a point in the

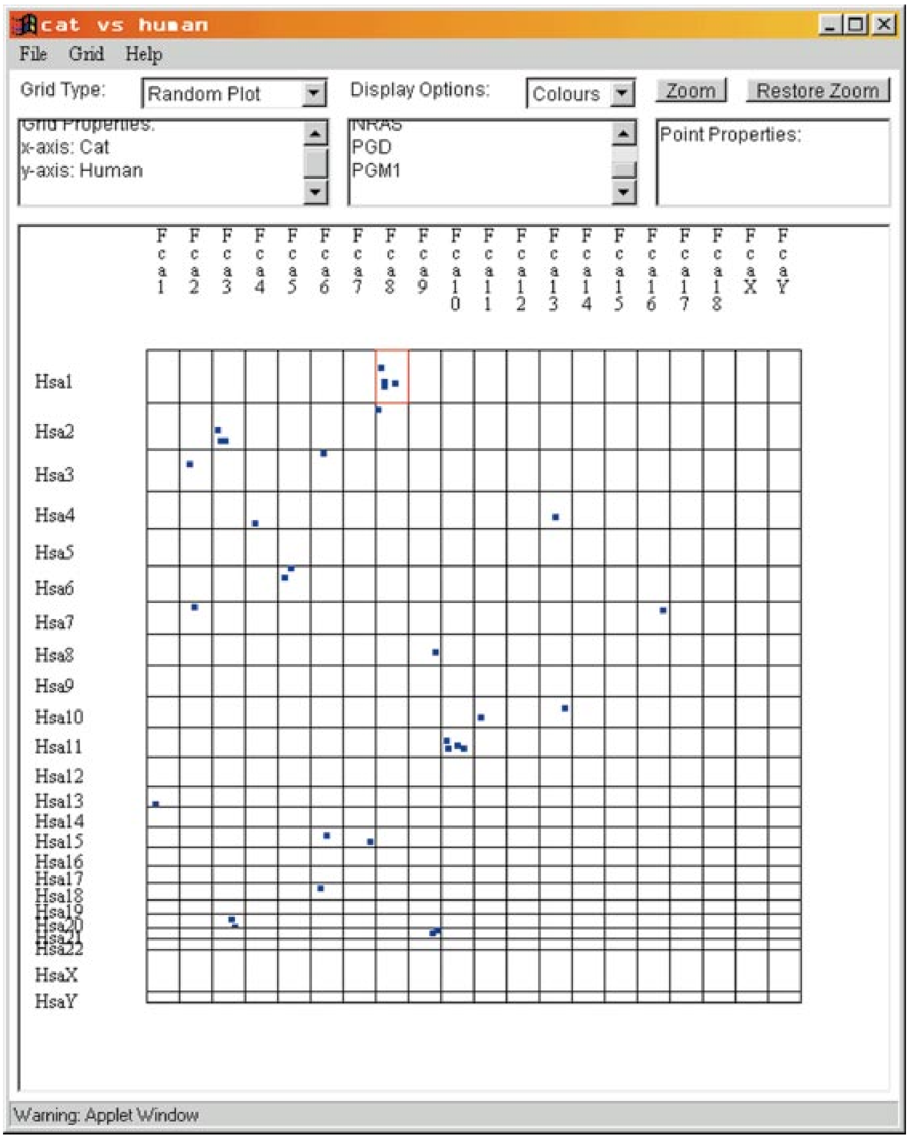

**Figure 1.** An Oxford Grid for Cat and Human using the Java tool GridMap and data from the ZooMap database. The cat chromosomes are plotted across the top of the grid and the human ones are down the side. The drop-down menus at the top allow users to select what type of grid is used for the plot and to edit the font of the text or the colours used for the plot. The zoom button allows the user to pick a cell on the grid and zoom in to see it in detail. The restore button returns to the whole grid

Copyright © 2000 John Wiley & Sons, Ltd.

cell causes the ‘point properties’ window to display the names of the genes that the point signifies.

The Pairwise Comparative Map tool (PCM) is meant to be downloaded by users for use with their own programs. A demonstration and detailed instructions for those wishing to download the tool are given. This Java bioWidget can be used to compare two genetic maps, drawing lines between homologous loci on the maps (Figure 2). It is designed to cope with genes present in only one map and with genes having two homologues on one map. The user is able to resize or invert either map to improve the comparison and each gene in the map can be linked to its entry in a sequence database. A more complex version, MultiPCM, can be used to display several PCMs in one image. Colour coding is used to indicate data from the same source and highlighting a chosen locus causes the locus to be highlighted in each PCM.

The Comparative Physical and Genetic Map tool (CPG Map) was developed by UK CropNet members working at IGER. It is a Java tool for displaying comparisons between physical and genetic maps. The view can be magnified, customized and zoomed. Magnification of just one of the maps can be done to allow viewing of more detail, in contrast to zooming, which can only be applied to both maps in the view. Customization options

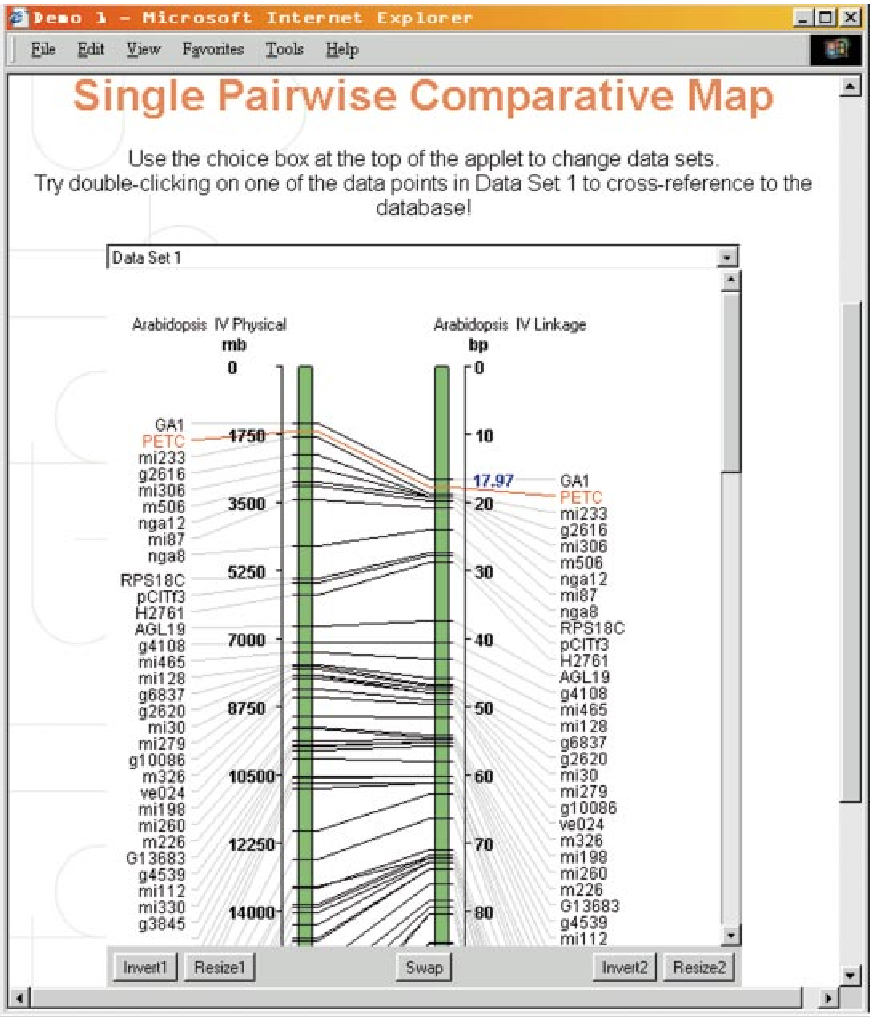

**Figure 2.** A demonstration of the Pairwise Comparative Map tool (PCM). Clicking on a locus of interest highlights it, and any homologues in the other map, in red. In this case, PETC has been highlighted. Buttons at the bottom of the window allow the user to invert or resize either map and each locus is linked to its database entry

Copyright © 2000 John Wiley & Sons, Ltd.

include colouring or resizing of items of interest in the map. The site includes a comprehensive demo, which I would encourage interested readers to try out for themselves.

The Genome Map Viewer is made available through the John Innes Centre Bioinformatics site. This is a comparative mapping tool that allows the user to compare plant genomes, drawn as concentric circles. To draw a map, choose 'import' from the import/export menu. Then select the source database and finally the organisms whose genomes you wish to plot. The map is then drawn with each genome coloured differently as concentric circles (Figure 3). The Display menu allows users to determine which features of the map they wish to be shown, such as telomeres and centromeres and the names of the homology blocks. Clicking on a block generates a smaller Applet window with details of the genome and chromosome from which the block is derived, its location in the map and, occasionally, extra notes such as a comment on the quality of the map in that region or the orientation of the block. The program is supported by a Users' Guide, which is made available from the same page.

### Databases

This page provides access to five UK CropNet databases, mirrors of 11 US plant databases and

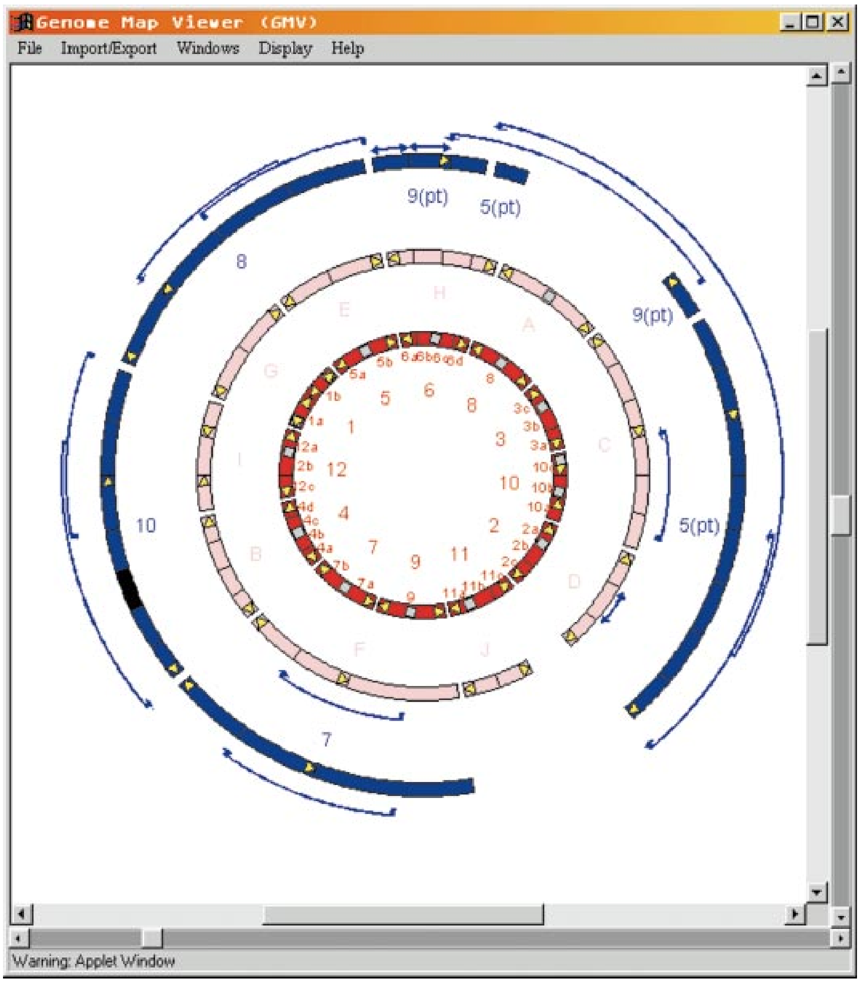

**Figure 3.** A Genome Map Viewer result for *Zea mays* (maize), *Sorghum bicolor* and *Orysa sativa* (rice), using data from the prototype comapDB. The outer blue circle is the maize genome, the pink circle is the *Sorghum* genome and the inner red circle is the rice genome. The grey boxes denote centromeres and the yellow triangles mark the telomeres. The blue arrows indicate possible translocations and inversions and the numbers are the names of the homology blocks two John Innes Centre databases, using either of two interfaces, AceBrowser and WebAce (Figure 4). The left-hand column of 'Database' links point to the project page for each UK CropNet database or to the home page of the US and John Innes Centre databases. The links in the right-hand columns provide access to the databases using either interface. The two interfaces differ only slightly in their style and the tools available. I chose to take the advice of the site authors and have reviewed the more intuitive AceBrowser interface.

The AceBrowser interface offers a search of a huge selection of subsections within any chosen database, for example, the *Arabidopsis* Genome Resource can be searched across categories such as *Arabidopsis* protein, gene name, locus, author and clone (Figure 5). The results are tabulated and each is a link to more information on that entry. Alternatively, users can make a search across all categories using the 'text search' button (Figure 6). It is also possible to browse the entire contents of any of these subsections of the data, by selecting it and carrying out a search without specifying a search term.

The RiceGenes database contains a quantitative trait loci (QTL) category, since many of these markers have been placed on the map of this

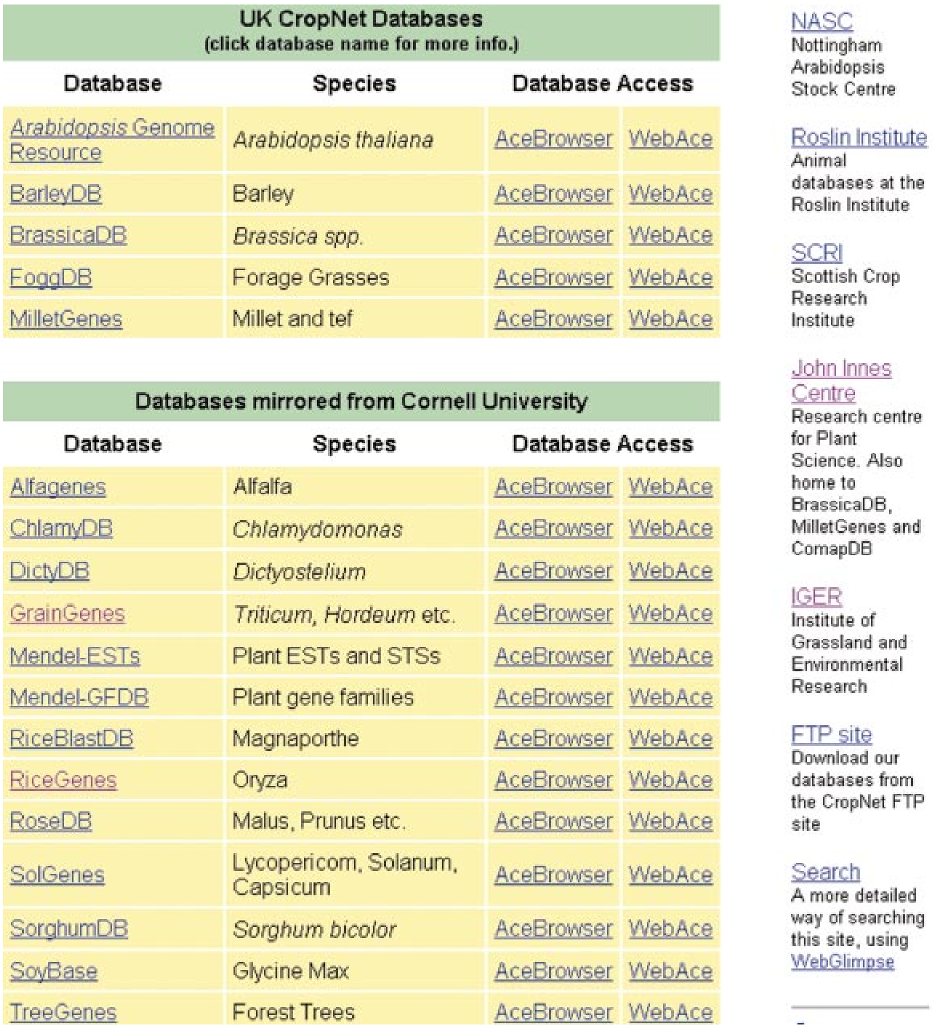

UK CropNet Databases

(click database name for more info.)

| Database | Species | Database Access | |
|---|---|---|---|
| Arabidopsis Genome Resource | *Arabidopsis thaliana* | AceBrowser | WebAce |
| BarleyDB | Barley | AceBrowser | WebAce |
| BrassicaDB | *Brassica spp.* | AceBrowser | WebAce |
| FoggDB | Forage Grasses | AceBrowser | WebAce |
| MilletGenes | Millet and tef | AceBrowser | WebAce |

Databases mirrored from Cornell University

| Database | Species | Database Access | |
|---|---|---|---|
| Alfagenes | Alfalfa | AceBrowser | WebAce |
| ChlamyDB | *Chlamydomonas* | AceBrowser | WebAce |
| DictyDB | *Dictyostelium* | AceBrowser | WebAce |
| GrainGenes | *Triticum, Hordeum* etc. | AceBrowser | WebAce |
| Mendel-ESTs | Plant ESTs and STSs | AceBrowser | WebAce |
| Mendel-GFDB | Plant gene families | AceBrowser | WebAce |
| RiceBlastDB | Magnaporthe | AceBrowser | WebAce |
| RiceGenes | Oryza | AceBrowser | WebAce |
| RoseDB | Malus, Prunus etc. | AceBrowser | WebAce |
| SolGenes | Lycopericom, Solanum, Capsicum | AceBrowser | WebAce |
| SorghumDB | *Sorghum bicolor* | AceBrowser | WebAce |
| SoyBase | Glycine Max | AceBrowser | WebAce |
| TreeGenes | Forest Trees | AceBrowser | WebAce |

NASC
Nottingham Arabidopsis Stock Centre

Roslin Institute

Animal databases at the Roslin Institute

SCRI
Scottish Crop Research Institute

John Innes Centre

Research centre for Plant Science. Also home to BrassicaDB, MilletGenes and ComapDB

IGER
Institute of Grassland and Environmental Research

FTP site

Download our databases from the CropNet FTP site

Search

A more detailed way of searching this site, using WebGlimpse

**Figure 4.** The databases provided by UK CropNet. These are grouped into UK CropNet databases or mirrors of databases held at Cornell University. Links are offered to the home page of each database (left column) and access to the database via AceBrowser or WebAce are offered (right columns)

Copyright © 2000 John Wiley & Sons, Ltd. 

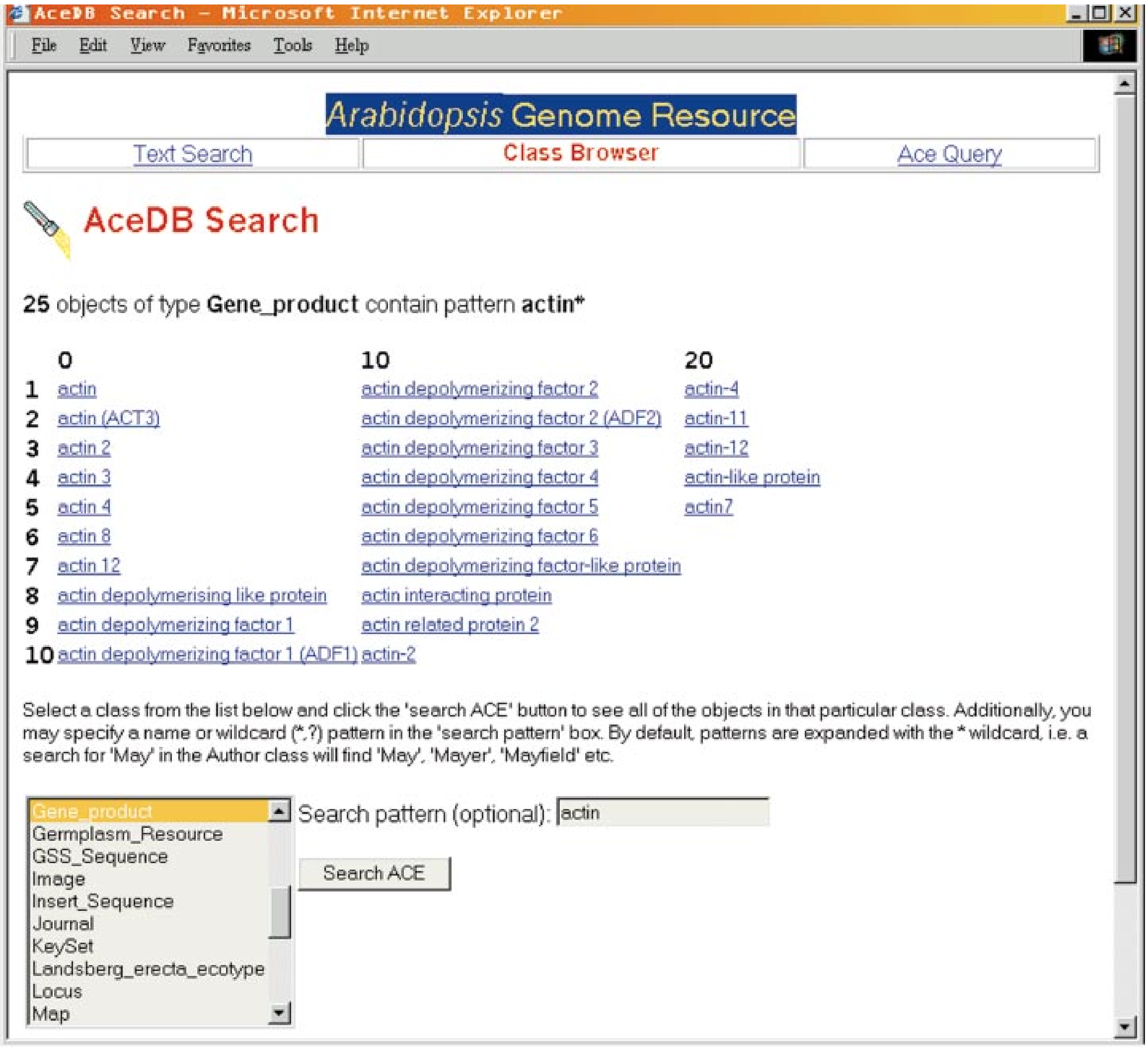

**Figure 5.** The results of a query of the 'gene product' section of the *Arabidopsis* Genome Resource (AGR) using the search term 'actin'. This returns a list of links to gene product entries for all gene products containing actin in their names important crop. Selecting a QTL from the list (Figure 7) returns a table of information, including the name of the trait, a list of markers giving significant linkage to the trait and the studies in which the trait was measured. All of these are links to further information, for example, clicking on the trait name (Figure 8) gives a description of the trait, provides an explanation of how it was measured and provides links to studies that assessed the trait and further trait information. ACeDB was designed with mapping in mind, so graphical display of the information is a strong point of the system. The 'graphic display' link appears at the top right corner of the page for every entry, and it is shown underlined and in blue if there is a graphic available for the item you are researching. Clicking on the link leads to a map of the region including the locus, gene or QTL of choice (Figure 9). In the case of a QTL query, this map includes a representation of the chromosomal block, the QTL(s) in the region of interest and the markers that have been placed on the map, highlighting those with linkage to the trait. Clicking on a locus redraws the graphic display, with items coloured to show their relationship to that locus. The map view can be zoomed in or out and there are several other options available to modify the display, although these do take some time to function.

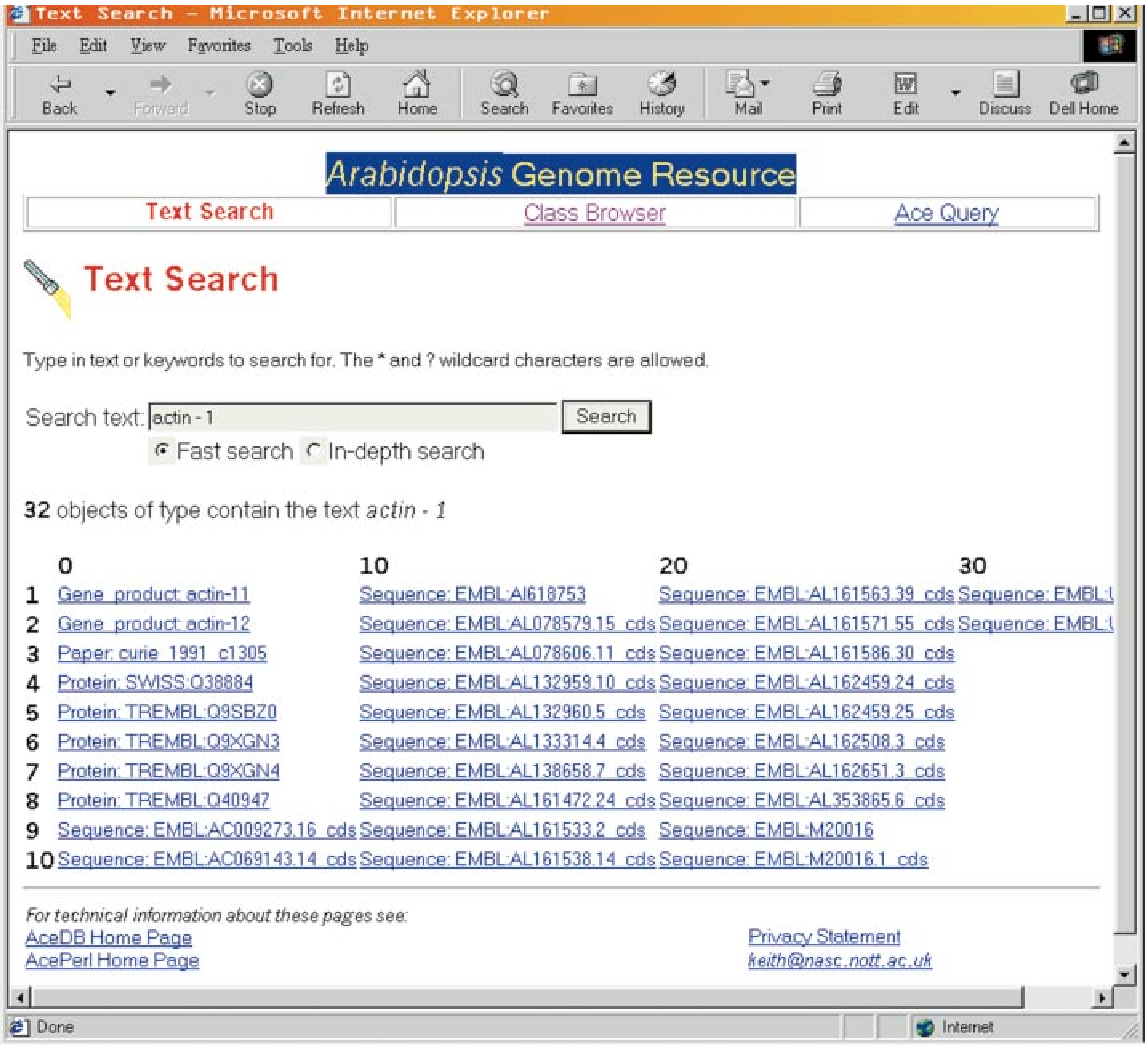

**Figure 6.** The results of a fast search using the 'text search' tool, with actin-1 as the query term. This tool searches across all sections of the database, in this case retrieving entries from the 'gene product', 'paper', 'protein' and 'sequence' sections. The result list is grouped by category

### What's new

This page has news of improvements and update reports for the site. This is used to explain (and draw to the attention of users to) any expansion of the databases or tools available at the site.

### Links

On the home page, this area provides links to the home pages of the institutes hosting the project teams and other related sites, such as those relating to ACeDB. There is also an FTP site where users can download some of the tools available from CropNet and a WebGlimpse search of the site. On other pages of the site, the content of this area changes to include links relevant to the contents of each page.

### Summary

Navigation around the site is made easy by having links to all of the pages, in the title bar of every page. Included in the title bar are a pull-down menu for quick access to the databases and a search of all the local pages. Each page also provides the

Copyright © 2000 John Wiley & Sons, Ltd.

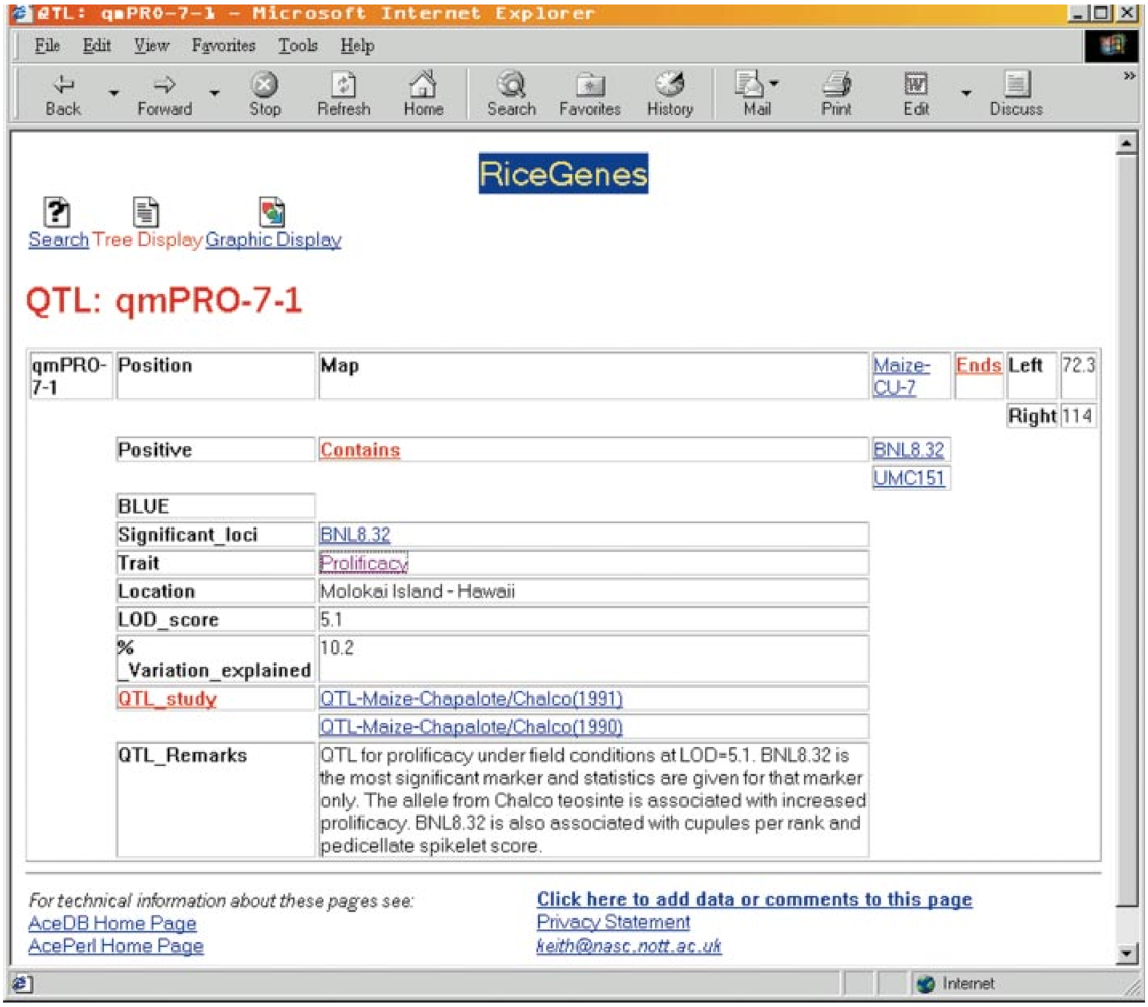

**Figure 7.** An example result of a QTL entry of the RiceGenes database. This includes information on markers showing linkage to the trait and links to information on the trait and to a map of the region around the QTL

opportunity for users to post their queries and feedback comments to Keith Bradnam, the Crop-Net webmaster, under the 'contact us' link.

The array of databases currently available is impressive, and the mirrors of the US sites provide faster access and permit cross-querying of this data for European users. The AceBrowser 'text search' tool is particularly useful for gleaning all the information on, for example, a chosen gene of interest.

The site has huge potential and will soon become an invaluable resource when the planned European databases (CerealsDB, ComapDB and SpudBase) and software tools (GFace, ARCADE and QAD genetic mapping) come on-line. The site is soon to be transferred to a new Compaq Alpha server (whilst retaining the same URL), which will allow mirroring of 18 databases from the Cornell site and possibly other ACeDB databases, such as LupinDB. The site authors are keen to hear from anyone who maintains a plant-related database in ACeDB format, with a view to mirroring their databases.

### Equipment details

This review was completed using a Dell PIII Inspiron 3700 Laptop, running Windows 98, with a permanent 10 Mbps Ethernet/Internet connection and a screen with 1024 × 768 pixels resolution. The

Copyright © 2000 John Wiley & Sons, Ltd.

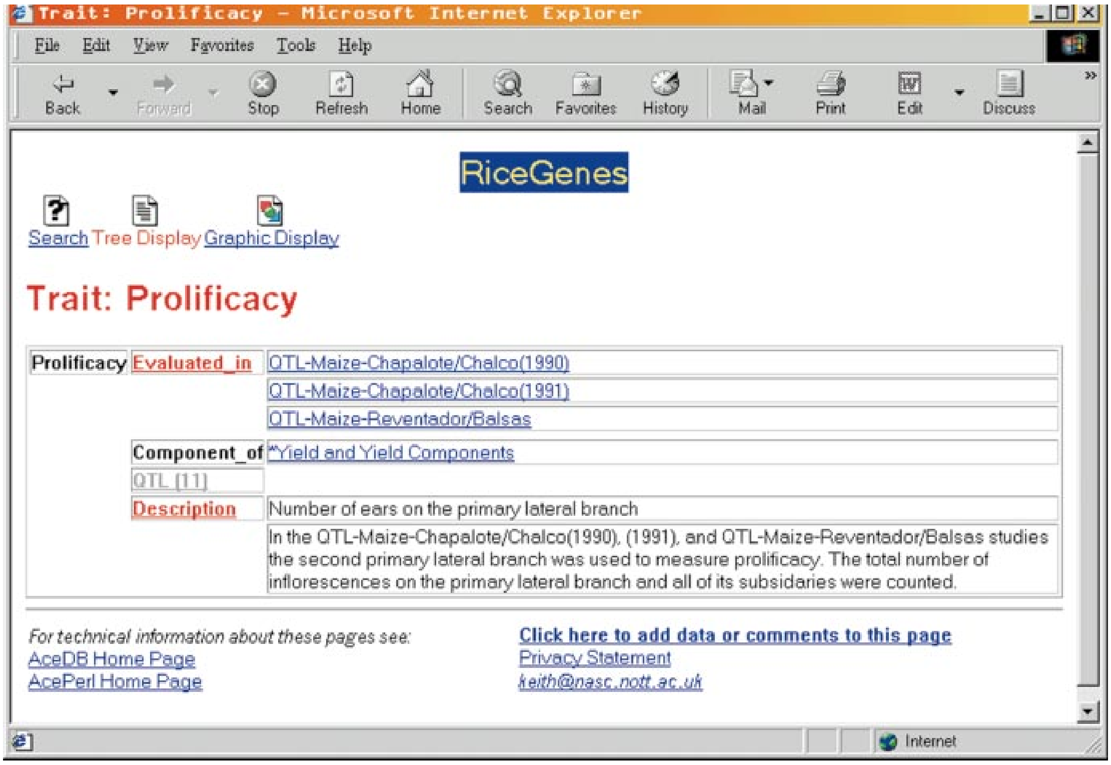

**Figure 8.** A typical example of a page giving details of a trait. Links are offered to each of three studies in which this trait has been measured and to information on the more complex trait of which prolificacy is a part. An explanation of the trait and how it has been measured in the studies is also given primary software used was Internet Explorer (IE), version 5. The AceBrowser pages of the site state that IE or Netscape version 4 or higher are required and users certainly do need Java-enabled browsers to use the site to its full potential.

### Mirror sites

**http://ars-genome.cornell.edu/** – 'Demeter's Genomes'. Several of the databases offered at UKCropNet are mirrors of databases held at this Cornell University site.

### Related sites

**http://www.ars.usda.gov/**
The Agricultural Research Service of the US Department of Agriculture (ARS).

**http://www.york.ac.uk/res/garnet/garnet.htm**
Garnet: Genomic *Arabidopsis* Resource Network.

**http://www.iger.bbsrc.ac.uk/igerweb/**
The Institute of Grassland and Environmental Research.

**http://www.jic.bbsrc.ac.uk/welcome.htm**
The John Innes Centre Bioinformatics Research Group.

**http://nasc.nott.ac.uk/**
The Nottingham *Arabidopsis* Stock Centre.

**http://www.ri.bbsrc.ac.uk/bioinformatics/databases.html**
Roslin Institute Bioinformatics.

**http://www.scri.sari.ac.uk/**
The Scottish Crop Research Institute.

### References

Dicks J, Anderson M, Cardle L, *et al*. 2000. UK CropNet: a collection of databases and bioinformatics resources for crop plant genomics. *Nucleic Acids Res* **28**: 104–107.

Copyright © 2000 John Wiley & Sons, Ltd. 

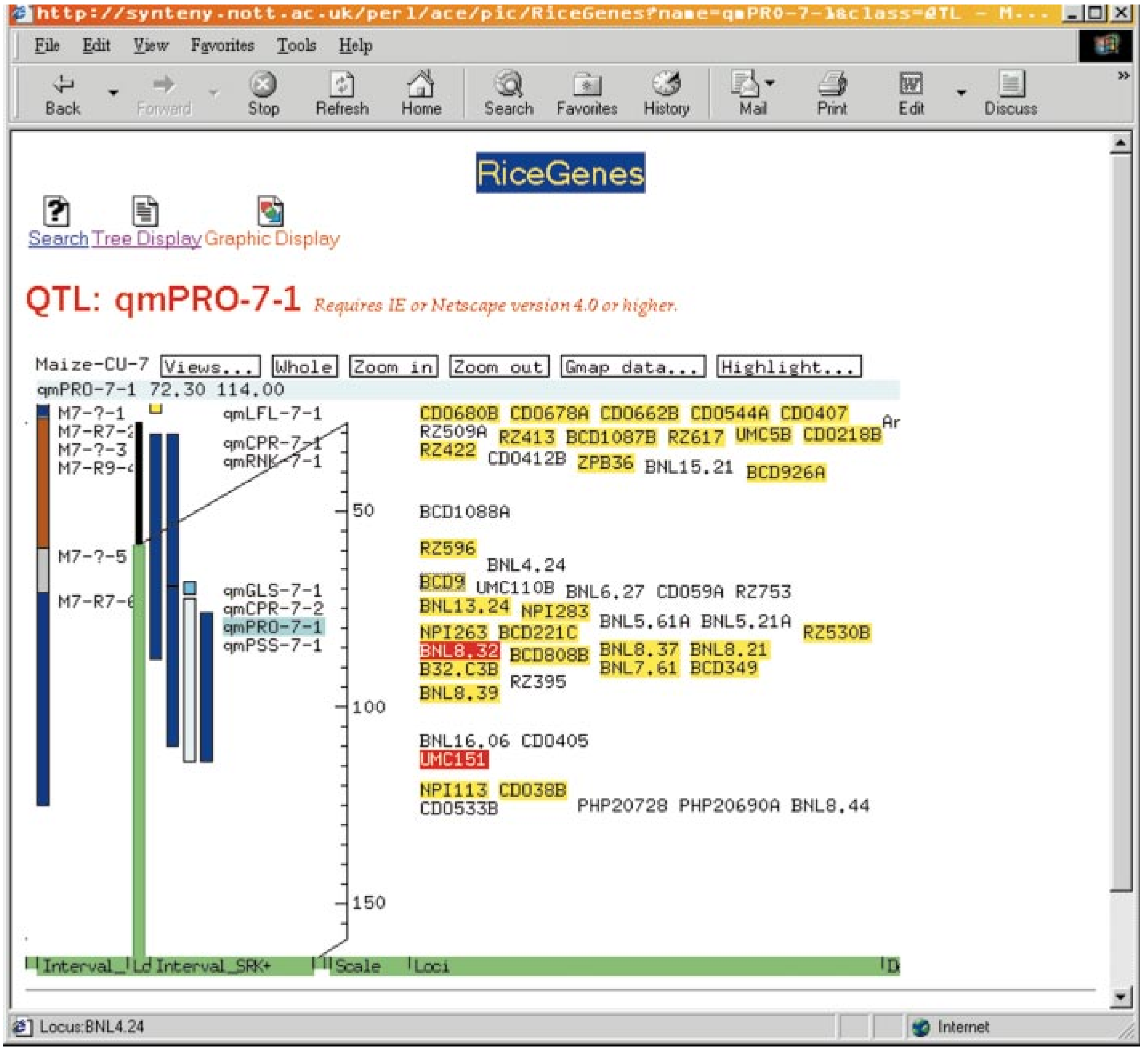

**Figure 9.** A graphical display for the QTL qmPRO-7-1. The left-most bar indicates the chromosome block in which the QTL maps; the green bar represents the active region in the display; the blue bars represent the QTLs mapped into this region, the QTL of interest being highlighted in pale blue. The text right of the grid lines indicates the loci mapping into this region, while those on a yellow background have been cloned. The two loci that have shown linkage to the QTL in question are picked out on a red background

---

Some of the sites reviewed will already be known to you but perhaps their content will be less well-known. The Website review is intended to help you discover new sites of interest, but also to provide a rapid and convenient means of revealing what you always knew was there but never had the time or inclination to look at. These articles are a personal critical analysis of the website. If you have any information about sites you think are worthy of being more widely known, the Managing Editor would be pleased to hear from you.

Copyright © 2000 John Wiley & Sons, Ltd.

UK CROPNET

### Announcement – External Training Sessions

UKcrop.net holds and represents nearly 20 public plant databases on a variety of model and crop species, and is developing and implementing a wide range of development tools for researchers in plant genomics and post-genomics. We would like to invite you to acquire some supervised tuition on the use of these tools in your own research.

Our current series of training sessions consist of mini-lectures and hands-on personal tuition by the developers and project leaders of UKcrop.net in an informal workshop setting at each of our development sites.

Potential and existing users of these international resources at beginner or advanced levels are all welcome.

### Calendar 2000/2001

**UKcropnet Training III**
Friday 1 December 2000, The John Innes Centre, Norwich.
Local Organizer: Jo Dicks.

**UKcropnet Training IV**
March/April 2001, SCRI, Dundee or NASC, Nottingham.
Date and venue to be confirmed.

For details see **http://ukcrop.net/training/**

Copyright © 2000 John Wiley & Sons, Ltd.
